# Domain-Aware Neural Architecture Search for Classifying Animals in Camera Trap Images

**DOI:** 10.3390/ani12040437

**Published:** 2022-02-11

**Authors:** Liang Jia, Ye Tian, Junguo Zhang

**Affiliations:** 1 School of Technology, Beijing Forestry University, Beijing 100083, China; sanctifier@cczu.edu.cn; 2 School of Microelectronics and Control Engineering, Changzhou University, Changzhou 213164, China

**Keywords:** camera trap images, convolutional neural network, neural architecture search

## Abstract

**Simple Summary:**

Camera traps acquire visual data in a non-disturbing and round-the-clock manner, so they are popular for ecological researchers observing wildlife. Each camera trap may record thousands of images of diverse species and bring about millions of images that need to be classified. Many methods have been proposed to classify camera trap images, but almost all methods rely on very deep convolutional neural networks that require intensive computational resources. Such resources may be unavailable and become formidable in cases where the surveillance area is large or becomes greatly expanded. We turn our attention to camera traps organized as groups, where each group produces images that are processed by the edge device with lightweight networks tailored for images produced by the group. To achieve this goal, we propose a method to automatically design networks deployable for edge devices with respect to given images. With the proposed method, researchers without any experience in designing neural networks can develop networks applicable for edge devices. Thus, camera trap images can be processed in a distributed manner through edge devices, lowering the costs of transferring and processing data accumulated at camera traps.

**Abstract:**

Camera traps provide a feasible way for ecological researchers to observe wildlife, and they often produce millions of images of diverse species requiring classification. This classification can be automated via edge devices installed with convolutional neural networks, but networks may need to be customized per device because edge devices are highly heterogeneous and resource-limited. This can be addressed by a neural architecture search capable of automatically designing networks. However, search methods are usually developed based on benchmark datasets differing widely from camera trap images in many aspects including data distributions and aspect ratios. Therefore, we designed a novel search method conducted directly on camera trap images with lowered resolutions and maintained aspect ratios; the search is guided by a loss function whose hyper parameter is theoretically derived for finding lightweight networks. The search was applied to two datasets and led to lightweight networks tested on an edge device named NVIDIA Jetson X2. The resulting accuracies were competitive in comparison. Conclusively, researchers without knowledge of designing networks can obtain networks optimized for edge devices and thus establish or expand surveillance areas in a cost-effective way.

## 1. Introduction

Visual data are a rich source of information about wildlife and can provide strong support for wildlife conservation and ecological research. One cost-effective way to obtain visual data of wildlife is via camera traps that work in a non-disturbing [1] and round-the-clock manner [2], thus making them ideal for observing wild animals otherwise difficult to monitor [3], e.g., nocturnal mammals [4] and large animals [5]. Because camera traps are noninvasive [6], a single deployment may record a diverse range of species [7]. Consequently, the recorded images have to be processed before being adopted in ecological research [8]. The images may go through several processing stages determined by the research process, a fundamental stage of which is species identification that is usually implemented as automatically and centrally classifying camera trap images at a data center installed with very deep convolutional neural networks (CNNs) [9,10,11,12,13]. In practice, there may be millions of images produced by camera traps [7,12,14,15], so image transfer and processing at a data center is often computationally intensive and costly. Furthermore, the scale of the surveillance area may also be restricted by the processing capability of the data center.

Edge computing [16] was ideally developed for such cases, i.e., intensive computation centralized at a data center can be split and localized by edge devices near camera traps [17,18]. Thus, fundamental processing steps such as removing images without animals [6,7,12,19,20,21] and classifying images with animals [9,10,11,12,13] can be automatically conducted on edge devices. However, edge devices are not only heterogeneous [22] but also resource constrained [23]. These limitations of edge devices narrow down the range of available neural networks [23,24]. Hence, lightweight networks [25] designed for edge devices are critical in edge computing for camera trap images. Even so, “deep neural network design is very difficult, and it requires the experience and knowledge of experts, a lot of trial and error, and even inspiration” [26]. Luckily, network design can be automated through neural architecture search (NAS) [27]. However, NAS is often developed regardless of domain knowledge [28] regarding camera trap images of wildlife [29]. Specifically, NAS is often designed based on benchmark datasets such as CIFAR-10 [30] and ImageNet [31], which differ from camera trap images in many aspects, especially data distribution and aspect ratios as described below.

The data distribution of benchmark datasets may differ from camera trap images, e.g., in classes, image foregrounds and backgrounds. Camera trap images purely contain animals, but only partial classes in benchmark datasets are relevant to animals. For instance, six out of ten classes in CIFAR-10 are related to animals and 233 out of 1000 classes in ImageNet are relevant to vertebrate [32]. Consequently, NAS based on benchmark datasets may waste resources on designing networks optimized for data irrelevant to animals. In addition to classes, animal images in benchmark datasets also differ from camera trap images in foregrounds and backgrounds. For benchmark datasets [30,31], images are usually artificially preprocessed to guarantee that foreground animals are large and centered and their backgrounds are relatively small and may differ from animal habitats in the wild. In contrast, animals in habitats are photographed by camera traps under various conditions, so the animals may appear at random locations in images and are often closely related with image backgrounds.

The image aspect ratios of benchmark datasets differ from camera trap images, e.g., the aspect ratios of CIFAR-10 and ImageNet are both 1:1 (the image width and height are the same), though this ratio may not hold for camera trap images. For instance, the resolutions of camera trap images range from 2048 × 1536 (aspect ratio: 4:3) to 2616 × 1472 (16:9) in North American Camera Trap Images, i.e., NACTI [13], and the resolutions range from 1920 × 1080 (16:9) to 2048 × 1536 (4:3) in Missouri Camera Trap Images, i.e., MCTI [33]. Therefore, networks found by NAS based on benchmark datasets may require that camera trap images be resized to satisfy the aspect ratio 1:1. However, resizing images may alter their aspect ratios and introduce interpolated pixels, often resulting in either misshaped animals or memory waste.

In short, images from benchmark datasets adopted by NAS often differ from camera trap images, and this difference potentially implies domain shift [34]. Additionally, it may be hard to modify existing networks in line with the applications [29]. These issues inspired us to develop NAS based on the domain knowledge of camera trap images for edge devices. We used the proposed method to conduct searches directly on camera trap images rather than images of benchmark datasets. The aspect ratios of camera trap images are maintained during the search, which is guided by a loss function particularly derived for finding the lightweight networks. The hyper parameter of loss function was theoretically analyzed and carefully chosen, and lightweight networks found by the search were tested on the NVIDIA Jetson X2 edge device. The experimental results confirmed the validity of the proposed method. The main contributions of this paper are as follows.

1. A method named Domain-Aware Neural Architecture Search (DANAS) was developed regarding the domain knowledge of camera trap images. The proposed method directly searches networks on camera trap images, thus avoiding negative effects such as the domain shift incurred by benchmark datasets in conventional search methods.
2. Aspect ratios of camera trap images are maintained during the search. As part of domain knowledge, the changes of aspect ratio may not be automatically tackled by neural networks. Therefore, the changes are manually eliminated by first finding the most frequent aspect ratio and then padding images whose aspect ratios differ from the most frequent one.
3. A loss function was derived to guide DANAS to find lightweight networks applicable for edge devices. A theoretical analysis of the proposed loss function was conducted, and the analysis revealed the value of hyper parameter in the loss function to boost its guiding effect on the search.

## 2. Materials and Methods

### 2.1. Datasets

Two datasets were employed in this study: MCTI and NACTI, containing 24 thousand and 3.7 million camera trap images, respectively, with varying resolutions. Since label errors are found in NACTI and its millions of images require too much computational resources, NACTI was selectively adopted in this study in the form of a subset named NACTI-a containing 29 thousand images with varying resolutions. The species data in NACTI-a and MCTI are illustrated in Table 1.

### 2.2. Method

**Figure 1:**
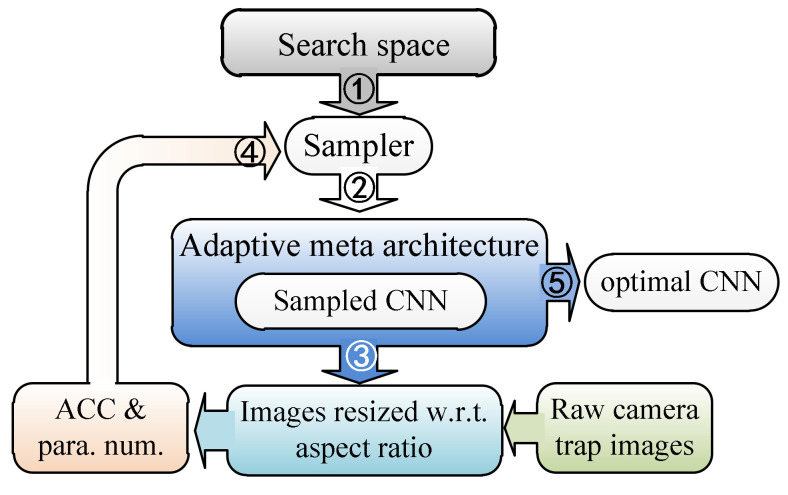
Flowchart of DANAS.

DANAS was developed within the framework of reinforcement learning [35,36], i.e., the search is implemented on sampling candidate networks from a search space through a sampler [29], as shown in Figure 1. In DANAS, the sampler is long short-term memory (LSTM) [37]. The reason to use LSTM as the sampler is that this sampler does not rely on parameter sharing [38], which may not be helpful for finding high-performance networks (as reported by [39]). Around the sampler, there are five conceptual search steps (from ① to ⑤ in Figure 1). By repeating these steps, the quality of the sampled network is gradually improved via updates of the learnable parameters θ of the sampler. Starting from the first step, all five steps are introduced sequentially next.

**Figure 2:**
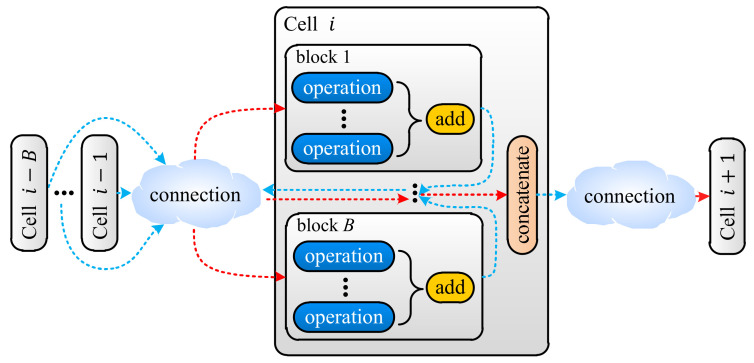
Meta normal cell.

In Step ① shown in Figure 1, LSTM samples candidate networks from the search space defined by a meta architecture, i.e., a prototype from which all candidate networks are derived. The meta architecture is similar to the ones defined in [35,36,37,38], i.e., a pipeline segmented to groups of layers called cells. There are two types of cells, normal and reduction cells, and the cells of the same type share the same inner structure. Besides the inner structures, the normal and reduction cells differ in the way they process data, i.e., the width and height dimensions of data remain the same before and after normal cells while the width and height of the input are halved through reduction cells. There are *N* normal cells in the pipeline, and each normal cell is adjacent to two reduction cells. At the end of the pipeline, a global average [40] is appended. In this study, the reduction cell was simplified to a single pooling layer, i.e., an average pooling or a max pooling with a kernel size of 5 × 5 or 3 × 3, and the normal cells were sampled based on the meta cell shown in Figure 2.

As shown in Figure 2, a normal cell is a group of blocks whose inputs come from blocks in the same cell or previous *B* cells. For blocks not serving as inputs of any other blocks, their outputs are concatenated to produce the cell output. Each normal cell has *B* (a constant) blocks, and each block has *M* (determined by the sampler) operations. The operation is sampled from the same set of operations as that in [38], e.g., a stack of 3 × 3 depth-wise-separable convolution [41], batch normalization [42], and ReLU [43]. Accordingly, the sampler first determines the operation number *M* of a block by sampling an integer from some predefined integers, and then it samples inputs and operations for the block, and the sampling repeats for *B* blocks to form a normal cell. Once the normal cell has been sampled, the sampler samples a pooling layer to form a reduction cell. Both the sampled normal cell and the reduction cell are employed to build the candidate network.

In Step ② shown in Figure 1, the candidate network is built based on the sampled cells and the meta architecture, i.e., assembling the cells according to the cell pipeline. The building process is identical with the one introduced in [44], i.e., we applied the adaptive meta-architecture [44] to build candidate networks. Once the candidate network is properly built, its performance is evaluated based on the camera trap images with maintained aspect ratios.

In Step ③ shown in Figure 1, the candidate network is trained and validated on camera trap images with the most frequent aspect ratio, i.e., the occurrences of unique aspect ratios of camera trap images are counted and the aspect ratio with the maximal count is chosen as the most frequent one. Images with aspect ratios different the most frequent one are padded by zero pixels. In practice, camera trap images are processed to have the same aspect ratio before the search starts, and the processed images are employed to train the candidate network. The trained network is then validated to yield validation accuracy to compute the loss.

In Step ④ shown in Figure 1, an accuracy reward [44] is generated based on accuracies obtained by training and validating a candidate network, and both the produced accuracy and the network parameter number [25] are employed to generate the loss J. The purpose of this step is to train LSTM to sample “good” networks via gradient-based optimization algorithms such as stochastic gradient descent (SGD). The meaning of “good” is twofold, i.e., the parameter number s of the network should be close to the desired parameter number ($s^{*}=1.5$ million in our case) and the accuracy reward R of the network should be close to the ideal accuracy ($R^{*}=100$, i.e., 100% accuracy). Since the reward is twofold, we need a bivariate reward function $f\left(R,s\right)$ so that the gradient $\nabla_{\theta }J$ of the total loss J synchronizes with the reward. According to the case of the unary loss function in studies of reinforcement learning [45], we defined $J\left(\theta \right)$ as

(1) $$J\left(\theta \right)=a_{\Sigma }\left(\theta \right)f\left(R,s\right),$$

where θ represents learnable parameters associated with the sampler, R is the accuracy reward involving the training and the validation accuracies of the candidate network, and s is the parameter number of the network in millions. The bivariate function $f\left(R,s\right)$ provides the reward based on $\left(R,s\right)$, and $a_{\Sigma }$ summarizes probabilities of sampling the candidate network through the sampler, i.e.,

(2) $$a_{\Sigma }\left(\theta \right)=\overset{n_{C}}{\underset{i}{\sum }}\overset{B_{i}}{\underset{j}{\sum }}\left(\log\left(P\left(n_{j}\left|\theta \right.\right)\right)+\overset{n_{j}}{\underset{k}{\sum }}\log\left(P\left(a_{k}\left|a_{1\;:\;\left(k-1\right)},\theta \right.\right)\right)\right),$$

where the notations are similar to [44], i.e., $n_{C}$ is the number of the cells in the candidate network, $B_{i}$ denotes the block number of the ith cell, $n_{j}$ is the operation number of the jth block, and $P\left(x\left|y\right.\right)$ is the probability of sampling x under condition y. The details of $a_{\Sigma }$ can be found in Appendix A. Since

(3) $$\nabla_{\theta \left|R,s\right.}J=f\left(R,s\right)\cdot \nabla_{\theta }a_{\Sigma },$$

and $f\left(R,s\right)$ yields a scalar, the direction of $\nabla_{\theta \left|R,s\right.}J$ is solely determined by $\nabla_{\theta }a_{\Sigma }$. However, $a_{\Sigma }$ remains unknown due to the unknown probability distributions of $n_{j}$ and $a_{k}$, which means the direction of $\nabla_{\theta }a_{\Sigma }$ is out of our control, i.e., we cannot change the direction of $\nabla_{\theta \left|R,s\right.}J$ to point to promising positions of high rewards. However, we can change its magnitude $\left|\nabla_{\theta \left|R,s\right.}J\right|$ via $f\left(R,s\right)$ so that $\left|\nabla_{\theta \left|R,s\right.}J\right|$ synchronizes with the reward. For example, suppose the sampler sampled a network of $\left(R,s\right)$ close to $\left(R^{*},s^{*}\right)$; we expect the sampler to sample networks alike, which requires that θ should not be largely updated by SGD involving $\left|\nabla_{\theta \left|R,s\right.}J\right|$. However, $\left|\nabla_{\theta \left|R,s\right.}J\right|$ is partially determined by $\left|\nabla_{\theta }a_{\Sigma }\right|$, so $\left|\nabla_{\theta \left|R,s\right.}J\right|$ may not remain small when $\left(R,s\right)$ is close to $\left(R^{*},s^{*}\right)$. In this case, $f\left(R,s\right)$ should scale $\left|\nabla_{\theta }a_{\Sigma }\right|$ to ensure that the resulting $\left|\nabla_{\theta \left|R,s\right.}J\right|$ is relatively small. This requires the reward surface defined by $f\left(R,s\right)$ to be similar to a whirlpool with vortex $\left(R^{*},s^{*}\right)$. We chose Witch of Agnesi [46] to build $f\left(R,s\right)$ on account of its bell-like curve and the simple mathematical form that only introduces one hyper parameter. Therefore, $f\left(R,s\right)$ is defined as

(4) $$f\left(R,s\right)={\left(R^{*}-\frac{8a^{3}R}{{\left(s-s^{*}\right)}^{2}+4a^{2}}\right)}^{2},$$

where a∈ℝ is the hyper parameter introduced by Witch of Agnesi. In practice, $R^{*}$ usually equals 100 (100% accuracy) [44], $s^{*}$ is determined by the application, and only a remains unknown. The value of a may be discovered by assuming both R and s are restrained within some range, and this assumption may be reasonable under certain search conditions. Specifically, let $x=s-s^{*}$ and y=R; then $f\left(R,s\right)$ can be written as

(5) $$f\left(x,y\right)={\left(R^{*}-\frac{8a^{3}y}{x^{2}+4a^{2}}\right)}^{2}.$$

Assuming $x_{1}\leq x\leq x_{2}$ and $y_{1}\leq y\leq y_{2}$, the volume V of $f\left(x,y\right)$ within the assumed ranges is given by

$V=\int_{y_{1}}^{y_{2}}\int_{x_{1}}^{x_{2}}{\left(R^{*}-\frac{8a^{3}y}{x^{2}+4a^{2}}\right)}^{2}dxdy$

$=\int_{y_{1}}^{y_{2}}\int_{u_{1}}^{u_{2}}{\left(R^{*}-\frac{2ay}{\tan^{2}u+1}\right)}^{2}d\left(2a\tan u\right)dy$

$=\int_{y_{1}}^{y_{2}}\int_{u_{1}}^{u_{2}}{\left(R^{*}-2ay\cos^{2}u\right)}^{2}\frac{2a}{\cos^{2}u}dudy$

$=\int_{y_{1}}^{y_{2}}\int_{u_{1}}^{u_{2}}\left(\frac{2aR^{*}{}^{2}}{\cos^{2}u}-4a^{2}R^{*}y+8a^{3}y^{2}\cos^{2}u\right)dudy$

$=\int_{y_{1}}^{y_{2}}\left(2aR^{*}{}^{2}\tan u\left|\begin{array}{c} {\;}^{u_{2}}\\ {\;}_{u_{1}} \end{array}\right.-4a^{2}R^{*}yu\left|\begin{array}{c} {\;}^{u_{2}}\\ {\;}_{u_{1}} \end{array}\right.+2a^{3}y^{2}\sin\left(2u\right)\left|\begin{array}{c} {\;}^{u_{2}}\\ {\;}_{u_{1}} \end{array}\right.+4a^{3}y^{2}u\left|\begin{array}{c} {\;}^{u_{2}}\\ {\;}_{u_{1}} \end{array}\right.\right)dy$where x=2atanu and $u_{1}=\tan^{-1}\left(\frac{x_{1}}{2a}\right)\leq u\leq \tan^{-1}\left(\frac{x_{2}}{2a}\right)=u_{2}$. Suppose $u_{2}=-u_{1}=u^{*}<\pi /2$ and $0\leq y\leq R^{*}$, then, the formula above can be simplified by substituting tanu and $\sin\left(2u\right)$ by their Taylor series of order three, i.e.,

(6) $$\begin{array}{l} V=\int_{y_{1}}^{y_{2}}\left(4aR^{*}{}^{2}\tan u\left|\begin{array}{c} {\;}^{u^{*}}\\ {}_{0} \end{array}\right.-8a^{2}R^{*}yu\left|\begin{array}{c} {\;}^{u^{*}}\\ {}_{0} \end{array}\right.+4a^{3}y^{2}\sin\left(2u\right)\left|\begin{array}{c} {\;}^{u^{*}}\\ {}_{0} \end{array}\right.+8a^{3}y^{2}u\left|\begin{array}{c} {\;}^{u^{*}}\\ {}_{0} \end{array}\right.\right)dy\\ =\int_{y_{1}}^{y_{2}}\left(4aR^{*}{}^{2}\tan u^{*}-8a^{2}R^{*}yu^{*}+4a^{3}y^{2}\sin\left(2u^{*}\right)+8a^{3}y^{2}u^{*}\right)dy\;\\ =\left(4aR^{*}{}^{2}\tan u^{*}\right)y\left|\begin{array}{c} {\;}^{R^{*}}\\ {}_{0} \end{array}\right.-4a^{2}R^{*}u^{*}y^{2}\left|\begin{array}{c} {\;}^{R^{*}}\\ {}_{0} \end{array}\right.+\frac{4}{3}a^{3}\left(\sin\left(2u^{*}\right)+2u^{*}\right)y^{3}\left|\begin{array}{c} {\;}^{R^{*}}\\ {}_{0} \end{array}\right.\;\\ \approx 4aR^{*}{}^{3}\left(u^{*}+\frac{u^{*}{}^{3}}{3}\right)-4a^{2}R^{*}{}^{3}u^{*}+\frac{4}{3}a^{3}R^{*}{}^{3}\left(4u^{*}-\frac{{\left(2u^{*}\right)}^{3}}{3!}\right)\\ =\frac{4}{3}aR^{*}{}^{3}\left(1-\frac{4}{3}a^{2}\right)u^{*}{}^{3}+4aR^{*}{}^{3}\left(1-a+\frac{4}{3}a^{2}\right)u^{*}\\ =\frac{4}{3}aR^{*}{}^{3}\left(\frac{3-4a^{2}}{3}u^{*}{}^{3}+\left(3-3a+4a^{2}\right)u^{*}\right),\; \end{array}$$

which is equivalent to

(7) $$u^{*}{}^{3}+\frac{3\left(3-3a+4a^{2}\right)}{3-4a^{2}}u^{*}=\frac{9V}{4aR^{*}{}^{3}\left(3-4a^{2}\right)},$$

which is a special case of monic cubic polynomials, i.e., the depressed cubic: $u^{*}{}^{3}+c_{1}u^{*}=c_{2}$. According to Cardano’s formula, the solution of the depressed cubic is

(8) $$u^{*}=\sqrt[{3}]{\frac{c_{2}}{2}+\sqrt[{2}]{\frac{c_{2}^{2}}{4}+\frac{c_{1}^{3}}{27}}}+\sqrt[{3}]{\frac{c_{2}}{2}-\sqrt[{2}]{\frac{c_{2}^{2}}{4}+\frac{c_{1}^{3}}{27}}}$$

where $c_{1}$ and $c_{2}$ are

(9) $$\left\{\begin{array}{c} c_{1}=\frac{3\left(3-3a+4a^{2}\right)}{3-4a^{2}}\\ c_{2}=\frac{9V}{4aR^{*}{}^{3}\left(3-4a^{2}\right)}\; \end{array}\right..$$

The solution $u^{*}$ of the depressed cubic requires

(10) $$\frac{c_{2}^{2}}{4}+\frac{c_{1}^{3}}{27}\geq 0,$$

which holds if $c_{1}\geq 0$. The numerator of $c_{1}$ is $3-3a+4a^{2}$ and its determinant is Δ<0, so $3-3a+4a^{2}>0$ holds regardless of a. The denominator of $c_{1}$ is $3-4a^{2}$, so $c_{1}\geq 0$ is equivalent to $3-4a^{2}>0$, which leads to $a^{2}<a<3/4$.

**Figure 3:**
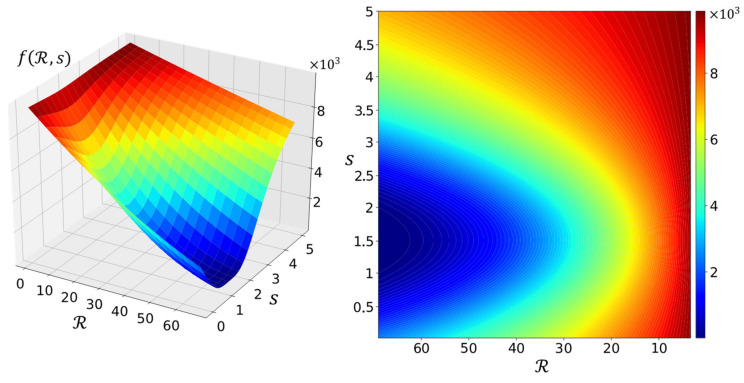
Surface of $f\left(R,s\right)$.

In practice, a=3/4−ε, where ε may take a small value such as $10^{-6}$. Figure 3 illustrates the surface of $f\left(R,s\right)$ parameterized by $a=3/4-10^{-6}$, $R^{*}=100$ and $s^{*}=1.5$ within the ranges $0\leq R\leq 2R^{*}/3$ and 0≤s≤5. As expected, f does have a whirlpool-like surface with the vortex $\left(R^{*},s^{*}\right)$, and the sampler may be guided by $\nabla_{\theta \left|R,s\right.}J$ involving f to find lightweight networks.

In step ⑤ shown in Figure 1, a selecting and training strategy is employed to find the optimal network. The idea behind this strategy is concentrating computational resources on promising networks found during the search, as the method first samples a relatively large number of candidate networks with small training epochs, e.g., 2 epochs in our case, and then finds the promising ones based on the sampled networks with large training epochs. In practice, we ran a single search to sample 1500 networks, and then networks with parameter numbers ranging from 1 to 1.5 million (the ideal parameter number in our case) were sorted decreasingly by their validation accuracies. If there are more than 150 networks, then only top 150 networks are retained for retraining through 5 epochs, and then the trained networks are sorted based on accuracies. If there are networks with accuracies >90%, then 15 networks are retained and retrained through 10 epochs; otherwise, half of the networks are retained and retrained. We stopped this procedure at 15 epochs and selected the top-1 network. If the difference between the accuracies between the top-1 and the top-2 networks was not large, e.g., less than 1%, then we would increase the epoch number and continue the training.

## 3. Results

The performance evaluation of DANAS was individually conducted on the NACTI-a and MCTI datasets. As shown in Table 1, the most frequent resolution of both NACTI-a and MCTI is 2048 × 1536 (aspect ratio 4:3). Accordingly, the images of the two datasets were resized to have the resolutions 85 × 64 (4:3) [44] for the search and 224 × 168 (4:3) for the test; for each dataset, the search was conducted on 85 × 64 images, and then the optimal network discovered by the search was trained and tested on 224 × 168 images. Each dataset was split to three subsets, i.e., the training set, the validation set and the test set, and the search was conducted on the first two subsets. The split was implemented by randomly sampling images from the dataset at a ratio of 0.64:0.16:0.2 of the sample numbers of three subsets, namely, 20% images were randomly sampled from the dataset to build the test set, then 20% images were randomly sampled from the remaining images to build the validation set, and the rest of the images served as the training set. The candidate networks found by the search were trained on the training set and then tested on the validation set, so the test set remained unknown to the search.

In searches on NACTI-a and MCTI, the pipeline shown in Figure 2 had three pairs of one reduction cell and five normal cells (N=3) at most. The normal cell had five blocks (B=5), and each block may have had five operations (M=5) at most. The input channels of the normal cell and reduction cell were, respectively, fixed to 20 and 40. The output channel of the reduction cell was fixed to 40, while the output channel of the normal cell was automatically determined by its operations. The candidate network was trained by using AMSGrad [47] with a batch size of 32, two epochs, and a learning rate of 0.005. The training was conducted on 85 × 64 training images via a PyTorch module named Distributed Data Parallel (DDP) that loaded the network and the batches to available GPUs, individually trained networks on GPUs, collected the resulting gradients from all GPUs and synchronized networks based on the collected gradients. The trained network was then tested on 85 × 64 images of the validation set on each GPU, and the resulting accuracies were retrieved via PyTorch module named Manager. The retrieved accuracies were then averaged to yield the training and the validation accuracies that were used to generate the accuracy reward. Finally, the loss was computed based on the accuracy reward through the loss function whose hyper parameters were set as $a=3/4-10^{-6}$, $R^{*}=100$ and $s^{*}=1.5$. All searches were done on a workstation installed with 4 GPUs of NVIDIA TITAN Xp, Ubuntu 20.04, PyTorch 1.7.0 and MySQL 8.0.13.

In tests, several networks famous for their lightweight designs or performance were chosen for comparison with DANAS, i.e., MobileNet-v2 [48], EfficientNet [49], DenseNet [50], Resnet-18 [51], ResNext [52] and Wide ResNet [53]. Each network was trained by using SGD [54] of Nesterov momentum [55] with a batch size of 10, 20 epochs, and a learning rate ranging from 0.005 to 0.0001. The learning rate was changed by cosine schedule [54]. The training was conducted on 224 × 168 images from both the training and the validation sets via DDP, and the weights of the network at the last epoch were saved on the hard disk. During tests, the weights were read from the disk and employed to populate the network, and the network was tested on 224 × 168 images of the test set. All networks in comparison were trained and tested on the workstation, and the optimal networks found by DANAS were additionally tested on an NVIDIA Jetson X2 edge device installed with Ubuntu 18.06 and PyTorch 1.1.0.

Since the camera trap images differ widely between MCTI and NACTI-a, DANAS found different networks, which led to distinct accuracies and misclassifications for two datasets. The detailed results are discussed in the following sections.

### 3.1. Search and Test on NACTI-a

**Figure 4:**
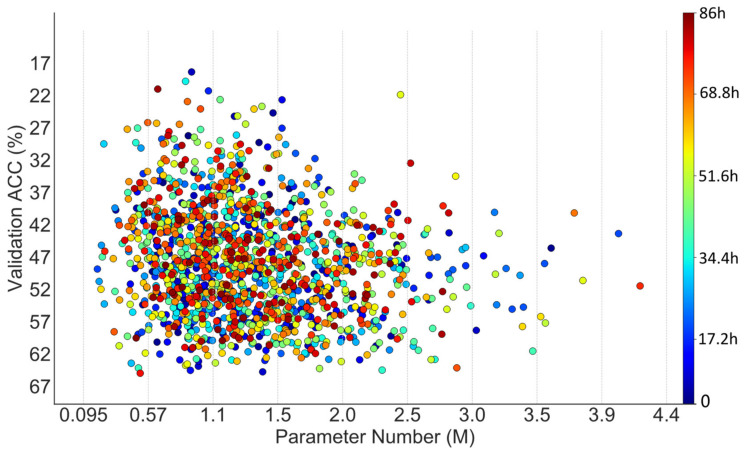
Scatter plot of parameter numbers and accuracies associated with the networks explored by a random search on NACTI-a.

**Figure 5:**
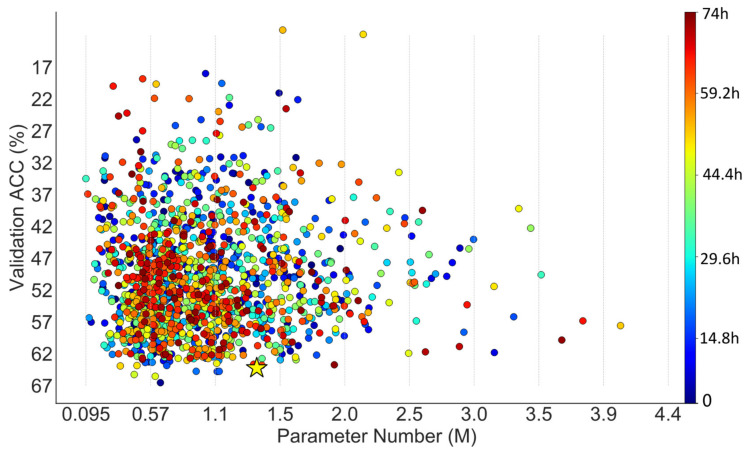
Scatter plot of parameter numbers and accuracies associated with the networks discovered by DANAS on NACTI-a.

The search on NACTI-a consumed roughly 74 hours and found a network with 1.36 million parameters. The search performance was compared with a random search via steps like those shown in Figure 1. Specifically, the sampler in step 1 of Figure 1 was replaced by random sampling, and both memory constriction [44] in step 2 and sampler updating in step 4 of Figure 1 were removed. However, the memory constriction could not truly be removed due to the limited physical GPU memory, and the constriction was thus alleviated by resampling the networks until the pipeline shrinkage [44] did not happen. The training and test configurations of networks explored by the random search were the same as those in DANAS. The search procedures of the random search and DANAS are visualized in Figure 4 and Figure 5, respectively.

As shown in Figure 4 regarding the random search, there were 57 networks with parameter numbers exceeding 2.5 million and 79 networks with validation accuracies exceeding 60%.

**Figure 6:**
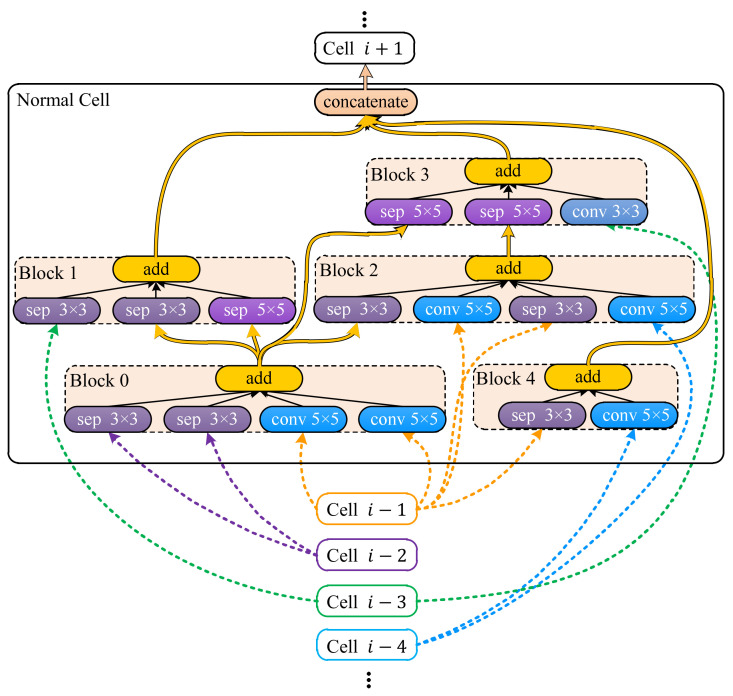
Normal cell found by DANAS on NACTI-a.

As shown in Figure 5 regarding DANAS, there were 32 networks with parameter numbers exceeding 2.5 million and 140 networks with validation accuracies exceeding 60%, and one of them was chosen as the optimal network according to step 5 in Figure 2. The optimal network is highlighted by a yellow star in Figure 5 and its normal cell is depicted in Figure 6; its reduction cell was simply a max pooling with a 3-by-3 kernel.

**Figure 7:**
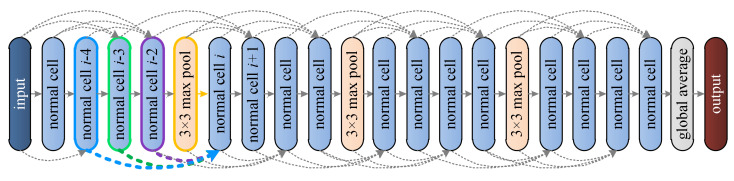
Network built based on the cell found by DANAS on NACTI-a.

The detailed network structure based on the normal cell shown in Figure 6 is illustrated in Figure 7, which shows how the data flowed through the normal and the reduction cells. The connections between cells are denoted by arrows. In Figure 7, cells labeled “normal cell i−4”, “normal cell i−3” …“normal cell i+1” correspond to cells labeled “Cell i−4”, “Cell i−3” …“Cell i+1” in Figure 6. 

If we rotate Figure 6 clockwise by 90°, then cell labels and arrow colors in Figure 6 will match labels and arrow colors in Figure 7. For instance, yellow arrows between “cell i−1” and “normal cell” in Figure 6 correspond to the yellow arrow between “3×3  max pool” and “normal cell i” in Figure 7, purple arrows between “cell i−2” and “normal cell” in Figure 6 correspond to the purple arrow between “normal cell i−2” and “normal cell i” in Figure 7, and so forth. For each normal cell shown in Figure 7, its inputs are signified by “a direct arrow running from the previous cell” and “three curved arrows running from another three previous cells”, and each arrow in Figure 7 corresponds to a group of arrows with the same color in Figure 6.

The input channels of the normal and reduction cells were fixed to 20 and 40, respectively. The output channel of the reduction cell was fixed to 40, and the output channel of the normal cell was automatically determined by its operations. The fixed channel numbers served as element-wise additions within blocks, i.e., only tensors of the same dimensions could be added element-wise. Therefore, channels of any block input were assumed to be 20. If the input channel differed from this constant, then the input was fed to an additional stack of 1-by-1 convolution, batch normalization and ReLU for changing the channel number to 20. Accordingly, the channel numbers of all block outputs were the same, i.e., 20, due to the fact that no operation within a block affected input data dimensions. Besides the channel numbers of inputs, if an input to a block differed in widths or heights, then all inputs were resized to have the minimal width and height found among the block inputs. Therefore, all block inputs shared the same dimensions, and element-wise additions worked in any block. As shown in Figure 6, the cell output was obtained by concatenating block outputs, which required that outputs to concatenate had the same width and height. If the outputs differed in width or height, then they were resized to the maximal width and height found among outputs to concatenate. The number of cell output channels could thus be easily derived by counting the number of outputs to concatenate, e.g., for the normal cell in Figure 6, its output channel number was 60=3×20, i.e., three block outputs were concatenated to yield the cell output. For reduction cell, since the pooling layer only halved the width and height dimensions of inputs, the output channel was the same as the input channel, i.e., 40. If inputs of a reduction cell had different channel numbers other than 40, then the inputs were fed to the channel-changing stack the same as the normal cell. All convolutions in normal cells had strides set to 1 and paddings set to 1 or 2, respectively, for 3×3 or 5×5 convolutions. All poolings had strides set to 2 and paddings set to 1 or 2, respectively, for 3×3 or 5×5 poolings.

As shown in Figure 7, the output of the last normal cell was fed to a global average, i.e., a w×h average pooling where w and h refer to the input width and height, respectively. Here, a w×h×c tensor was pooled to c scalars via the global average where c denotes the class number. If the input to the global average had channels other than c channels, then the input was fed to an additional 1-by-1 convolution of stride set to 1 and padding set to 0 before the input was fed to the global average. The results of the network shown in Figure 7 are illustrated in Table 2, and the best accuracy within each row is highlighted by bold texts.

As shown in Table 2, although the parameter number of the optimal network discovered by DANAS was small, the average test accuracy associated with DANAS was the third best of the compared networks. However, there were eight species accuracies in DANAS that were the best (bold digits in Table 2) compared to other networks, and there were eight best species accuracies in Resnet-18, which demonstrated the best average test accuracy.

**Figure 8:**
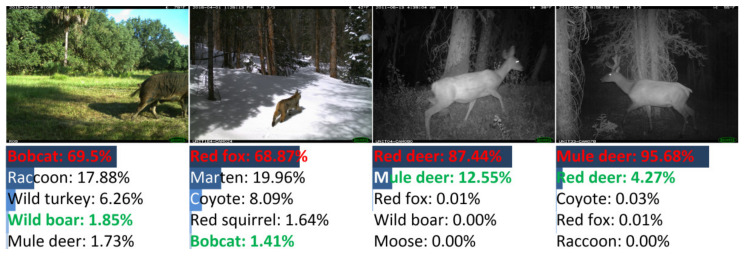
Examples of misclassified images from NACTI-a.

There were 155 images misclassified by DANAS. Among all misclassifications, 78 were color images and the rest were night-vision images, i.e., about half misclassified images were night-vision images. The image samples of typical misclassifications are illustrated in Figure 8, i.e., the partial animal body in the left sample, the small region occupied by the animal in the middle left sample, and visually similar animals in the right and the middle right samples.

**Figure 9:**
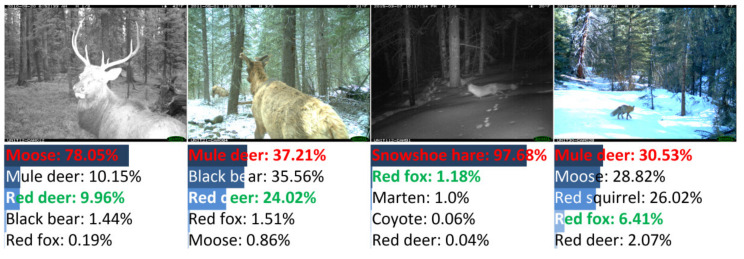
Samples of misclassified deer images in NACTI-a.

Among all misclassifications, about 64% (99 samples) were misclassified due to the visual similarity of animals, and these misclassifications mainly originated from the deer and canine species. Samples of deer and canine misclassifications are shown in Figure 9. The misclassifications were mainly made among red deer (29 samples) and red fox (23 samples). For red deer samples, 14 samples were grayscale images without colors (the left sample in Figure 9), and the remaining color samples always contained red deer whose heads were obscured due to camera view limitations, body orientations (the middle-left sample in Figure 9), etc. For red fox samples, 11 samples were grayscale images (the middle-right sample in Figure 9), and the remaining color samples always contained foxes occupying small image regions (the right sample in Figure 9).

**Figure 10:**
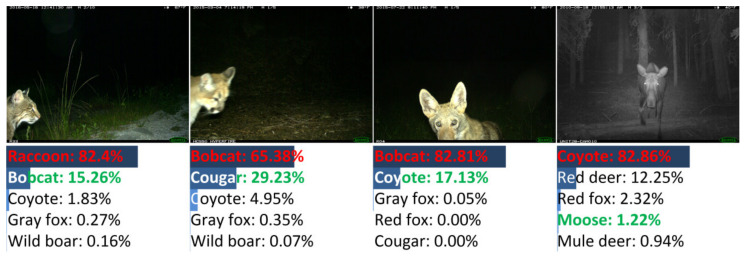
Samples of misclassified images of similar animals in NACTI-a.

Samples of misclassifications other than deer are shown in Figure 10. The misclassifications were made among bobcat, cougar, coyote, moose, etc., due to reasons similar to those of the deer and red fox misclassifications. Additionally, misclassification samples only containing animal heads are shown in Figure 10.

### 3.2. Search and Test on MCTI

**Figure 11:**
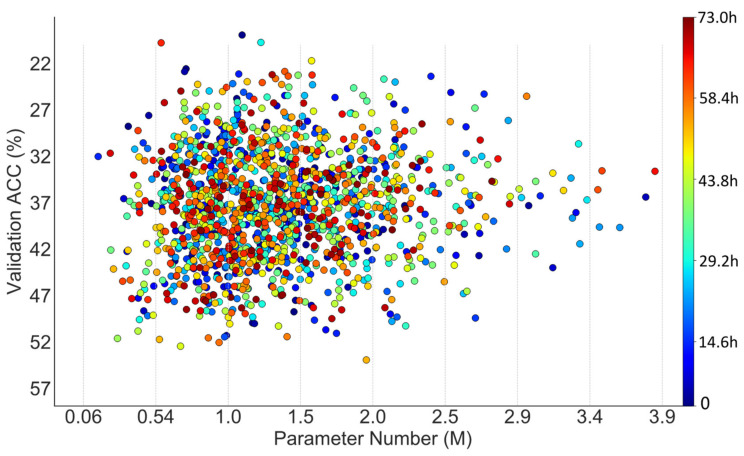
Scatter plot of parameter numbers and accuracies associated with the networks explored by a random search on MCTI.

**Figure 12:**
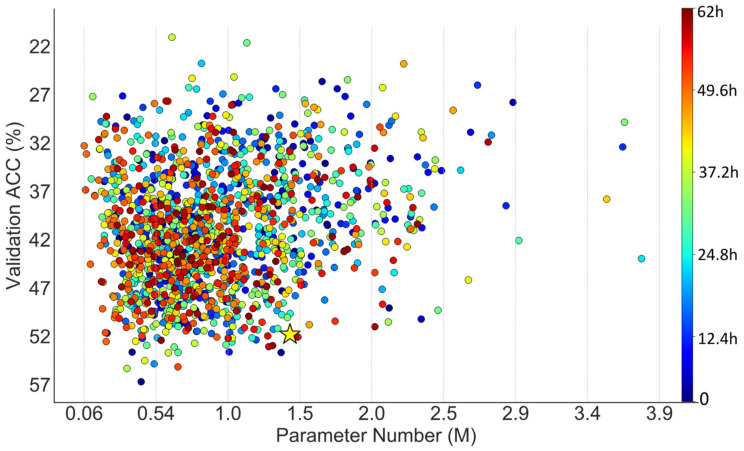
Scatter plot of parameter numbers and accuracies associated with the networks discovered by DANAS on MCTI.

The search on MCTI consumed roughly 62 hours and found a network with 1.43 million parameters. The search performance was compared with a random search whose configuration was the same as the one introduced in the previous section. The search procedures of DANAS and the random search are visualized in Figure 11 and Figure 12, respectively.

As shown in Figure 11 regarding the random search, there were 66 networks with parameter numbers exceeding 2.5 million (62 points on the right of vertical line at 2.5 in the figure; four points are not shown due to limited space) and 16 networks with validation accuracies exceeding 50%.

**Figure 13:**
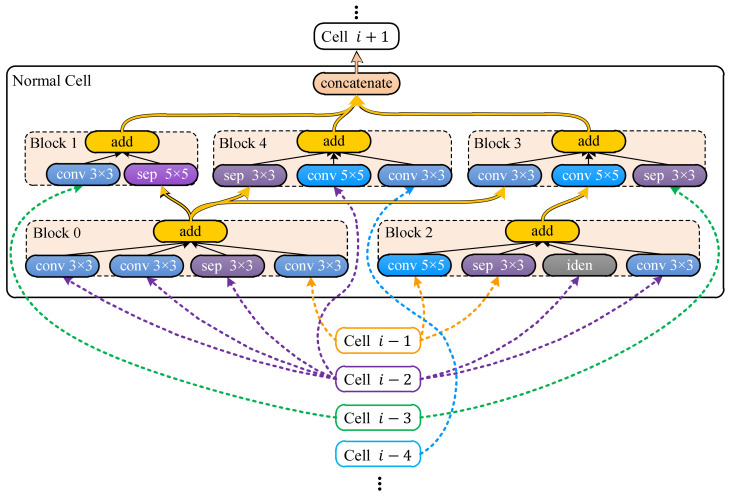
Normal cell found by DANAS on MCTI.

As shown in Figure 12 regarding DANAS, there were 15 networks with parameter numbers exceeding 2.5 million (13 points on the right of vertical line at 2.5 in the figure; two are not shown due to limited space) and 93 networks with validation accuracies exceeding 50%; one of them was chosen as the optimal network according to step 5 in Figure 2. The optimal network is highlighted by a yellow star in Figure 12, its normal cell is depicted in Figure 13; its reduction cell was simply a max pooling with a 3-by-3 kernel.

The network structure based on the cell in Figure 13 was the same as the one shown in Figure 7 because normal cells found on both NACTI-a and MCTI involved all previous cells, and the pipeline in Figure 7 illustrates how data flowed at the cell level (in contrast to the data flow at the block level that is shown in Figure 6 and Figure 13). The test results are shown in Table 3, and the best accuracy within each row is highlighted by bold texts.

**Figure 14:**
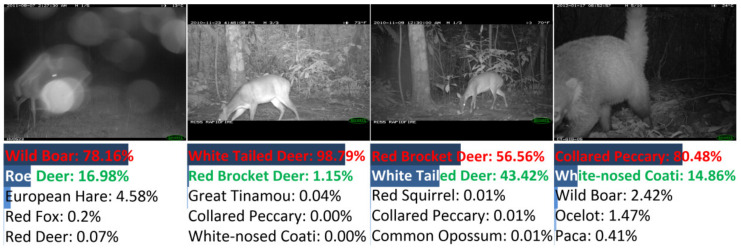
Examples of misclassified images from MCTI.

As shown in Table 3, the parameter number of the optimal network discovered by DANAS was small, and the average test accuracy associated with DANAS was the best throughout the networks in comparison. There were 167 images misclassified by DANAS. Among all misclassifications, 45 were color images, and the rest were grayscale images. Samples of typical misclassifications are shown in Figure 14, i.e., vagueness due to dirty camera lens in the left sample, similar backgrounds and species in the middle samples, and partial animal body in the right sample.

### 3.3. Tests on Jetson X2

**Figure 15:**
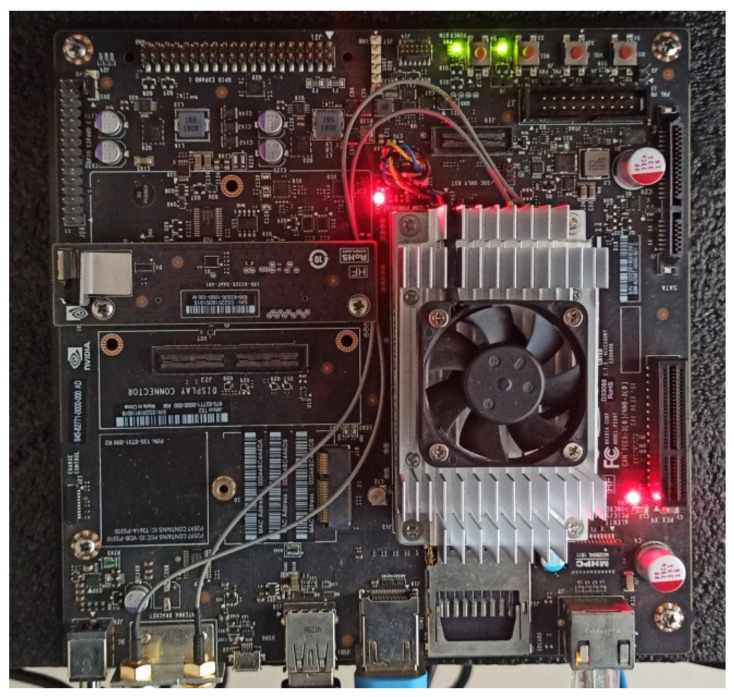
NVIDIA Jetson X2.

The optimal networks discovered by DANAS with the MCTI and NACTI-a datasets were tested on the NVIDIA Jetson X2 edge device shown in Figure 15. Because the versions of PyTorch installed in the workstation and the Jetson X2 are different, the format of network weights saved in the workstation was incompatible with Jetson X2. This issue was tackled by loading and resaving weights in Pickle-based format through PyTorch’s built-in function torch.save() with the parameter “_use_new_zipfile_serialization” set to False. The resaved network weights and 224 × 168 test images were copied to Jetson X2 through secure copy protocol as in [44]. The test results are shown in Table 4.

As shown in Table 4, the average accuracies on Jetson X2 were 92.91% and 94.31% for NACTI-a and MCTI, respectively. The average accuracies on Jetson X2 were slightly lower than the corresponding accuracies obtained on the workstation, i.e., 92.86% and 94.02% for NACTI-a and MCTI, respectively.

### 3.4. Comparisons between DANAS and other Search Methods

Since comparisons of search methods based on custom-defined search space and various hardware may introduce bias, we compared our method with other methods via Nasbench-201 [39]. Nasbench-201 provides a database and application programming interfaces (APIs) for comparing search methods with the same search space and hardware. In Nasbench-201, all candidate networks in a specific search space were trained, validated and tested on the CIFAR-10, CIFAR-100 and ImageNet-16-120 datasets [39]. The training, validating and testing accuracies were saved in databases and could be programmatically retrieved via an API, a network code encoding the network architecture. There were five operations scattering in three cells, i.e., the first cell containing one operation, the second containing two, and the last containing three. For each operation, there were five options available for sampling, i.e., “nor_conv_3 × 3”, “none”, “nor_conv_1 × 1”, “avg_pool_3 × 3” and “skip_connect” [39]. Nasbench-201 does not distinguish networks of different operation inputs (i.e., for all networks of operations arranged in the same encoding order, only one network is trained, validated and tested on the aforementioned datasets.), so there are $5\times 5^{2}\times 5^{3}=15,625$ [39] networks in Nasbench-201, and a sampler tested on Nasbench-201 is restricted to sample operations only. Accordingly, we simplified our sampler and applied Bayesian optimization [21] to automatically find values of the sampler hyper parameters, i.e., the embedding dimension, the hidden unit number, the layer number, and the learning rate were set to 19, 33, 1, and 0.005, respectively. The rest of the configuration was the same as that of DANAS.

According to [39], there are two types of search methods tested on Nasbench-201, i.e., methods dependent on or independent of parameter sharing. Parameter sharing often means weights of a newly-sampled network are initialized by using weights from the previously-sampled networks trained on the dataset, so the weights of previously trained networks are not abandoned during the search. In [39], parameter-sharing-dependent methods were repeated three times and other methods were repeated 500 times. For each run of the method independent of parameter sharing, the method continued to run until the simulated training time [39] of its sampled networks reached a predefined limit called time budget, i.e., 12,000 s. [39]. The simulated training time of the sampled network was obtained by adding its training and validation time saved in Nasbench-201.

Since our method (DANAS) is independent of parameter sharing, DANAS was tested according to the configuration of search methods independent of parameter sharing, i.e., the search based on DANAS was repeated 500 times and each search automatically stopped once the time budget was reached. Different from methods in [39], our method requires an additional hyper parameter, i.e., the ideal parameter number $s^{*}$. This parameter is set to the parameter number of the candidate network with the optimal validation accuracy. Accordingly, DANAS was tested against three datasets available in Nasbench-201, i.e., CIFAR-10 ($s^{*}=0.87$ in millions), CIFAR-100 ($s^{*}=0.86$ in millions), and ImageNet-16-120 ($s^{*}=1.29$ in millions). Because network weights required by parameter-sharing-dependent methods were not available at the time of paper submission, we only tested parameter-sharing-independent methods with Nasbench-201 on our own hardware. Specifically, all search steps except for training, validating and testing sampled networks were conducted on our hardware, and network accuracies and parameter numbers were directly retrieved from Nasbench-201. The configurations of all methods except DANAS were the same as [39]. The results are illustrated in Table 5.

As shown in Table 5, five search methods were compared on three benchmark datasets, i.e., CIFAR-10, CIFAR-100 and ImageNet-16-120 [39]. Among methods in comparison, i.e., REA [56], RS [57], REINFORCE [45] and BOHB [58], our method (DANAS) achieved the second best test accuracy on CIFAR-10 and the third best test accuracy on both CIFAR-100 and ImageNet-16-120.

## 4. Discussion

DANAS was evaluated on two datasets, NACTI-a and MCTI. For both datasets, the random searches significantly differed from DANAS in changes of validation accuracy and parameter number over time.

In the case of NACTI-a, the number of networks with parameter numbers exceeding 2.5 million in the random search was almost twice that of DANAS, and the number of networks with validation accuracies exceeding 50% in the random search was roughly half that of DANAS. More importantly, the distribution of points from DANAS in Figure 5 illustrates a growing trend towards networks with few parameter numbers and high validation accuracies, i.e., the search tended to find Pareto solutions good for both accuracy and parameter number, while no such trend can be seen in Figure 4 regarding the random search.

In the case of MCTI, the ratio between the numbers of networks with 2.5 million parameter numbers or above for random search and DANAS was higher than the case of NACTI-a, i.e., about 4:1, and the ratio between the numbers of networks with validation accuracies exceeding 50% for the random search and DANAS was lower than the case of NACTI-a, i.e., about 1:8. The distribution of points from DANAS in Figure 12 illustrates the same trend as the case of NACTI-a, and the random search showed no such trend, as depicted in Figure 11.

The performance of the networks found by DANAS was evaluated by comparing the test accuracies with seven CNNs with parameter numbers ranging from 0.7 to 66.8 million on two datasets, NACTI-a and MCTI. Although the parameter numbers of networks found on both datasets were lower than 1.5 M, the test accuracy was the third best for NACTI-a and the best for MCTI. These results reveal the benefit of designing CNNs with structures highly customized for studied data and used device. Generally, the experimental results confirmed the validity of DANAS.

The search efficiency of DANAS was compared with search methods reported in [39] based on Nasbench-201, and the search methods with parameter sharing were retested on our hardware. For all benchmark datasets of Nasbench-201, our method outperformed all parameter-sharing-dependent methods reported in [39] and most of parameter-sharing-independent methods including the random search. Generally, DANAS outperformed NAS methods with parameter sharing and was competitive compared with NAS methods without parameter sharing.

## 5. Conclusions

In this study, DANAS is proposed to automatically design lightweight CNNs for ecological research powered by camera traps and edge computing. DANAS was developed based on domain knowledge of camera trap images, i.e., the search is conducted on camera trap images whose resolutions are lowered while the original aspect ratios are maintained. Therefore, the data distribution of the original dataset is preserved during the search, so the data distribution difference incurred by using benchmark datasets in traditional NAS is reduced in DANAS. Furthermore, the search in DANAS is guided by a loss function designed based on Witch of Agnesi whose hyper parameter was theoretically derived. In experiments, DANAS was shown to successfully find lightweight networks for two datasets of wildlife camera trap images. The found networks were then trained on a workstation and tested on both the workstation and an edge device. In comparison with CNNs of classical lightweight designs and good performance, the networks found by DANAS had low parameter numbers and competitive test accuracies. Generally, researchers without knowledge of designing CNNs can obtain lightweight CNNs optimized for edge devices through DANAS and thus expand surveillance areas in a cost-effective way.

## Figures and Tables

**Table 1:** Dataset details.

Species in NACTI-a ^1^	2048 × 1536 (4:3)	1920 × 1080 (16:9)	2616 × 1472 (16:9)	Species in MCTI	2048 × 1536 (4:3)	1920 × 1080 (16:9)
Black bear ^2^	2420/534	10/1		Agouti	499/107	279/65
Marten ^2^	72/16			Bird	584/120	70/20
Red squirrel ^2^	313/75			Coiban Agouti	1135/245	18/2
Jackrabbit ^3^	594/135	55/8		Collared Peccary	372/83	398/85
Bobcat	2040/453	15/2		Opossum	454/94	295/73
California quail	277/60			European Hare	578/122	
Cougar	2380/527			Great Tinamou	681/148	380/66
Coyote	1416/322	55/14	6/1	Mouflon	1940/425	
Gray squirrel	811/186	1/0		Ocelot	256/64	184/35
Elk	1754/393	8/1		Paca	772/162	200/62
Gray fox	1253/279	5/2		Red Brocket Deer	425/94	384/78
Moose	978/216			Red Deer	2321/509	
Mule deer	1761/397			Red Fox	410/91	
Armadillo ^4^	521/113			Red Squirrel	343/78	182/36
Raccoon	1126/250			Roe Deer	1038/233	
Red deer	1754/374			Spiny Rat	383/91	201/37
Red fox	266/59			White-nosed Coati	883/192	179/41
Snowshoe hare	1183/263			White-tailed Deer	1363/287	452/106
Striped skunk	1080/243			Wild Boar	1538/345	
Virginia opossum	91/19			Wood Mouse	1105/245	
Wild boar	1548/340	4/2				
Wild turkey	643/155	17/0				

^1^ Numbers before and after slashes, respectively, refer to the training and testing image numbers; ^2^ American animals; ^3^ Black-tailed jackrabbit ^4^ Nine-banded armadillo.

**Table 2:** NACTI-a accuracy comparison.

Species or Parameter Number	DANAS (Ours)	MobileNet-v2 [48]	EfficientNet [49]	DenseNet [50]	Resnet-18 [51]	ResNext [52]	Wide_ResNet [53]	Random Search
Para. num.	1.36	2.25	4.04	6.98	11.19	23.02	66.88	**0.52**
Black bear ^1^	98.32	97.57	96.07	**99.44**	98.13	98.88	98.88	98.69
Marten ^1^	25.00	6.25	25.00	**62.50**	37.50	31.25	37.50	0.00
Red squirrel^1^	98.67	97.33	**100**	96.00	**100**	33.33	20.00	**100**
Jackrabbit ^2^	99.30	99.30	98.60	99.30	**100**	99.30	98.60	98.60
Bobcat	**97.58**	96.92	96.26	97.36	96.26	96.48	95.60	97.14
Quail ^3^	96.67	95.00	98.33	96.67	**100**	90.00	83.33	96.67
Cougar	98.10	96.20	96.20	**99.05**	98.48	95.26	95.83	97.53
Coyote	95.55	94.07	**95.85**	92.88	93.77	81.90	78.34	93.18
Gray squirrel ^4^	**100**	97.31	97.85	96.24	98.92	93.01	97.85	96.77
Elk	99.24	99.24	97.97	**99.75**	99.49	98.98	98.98	99.49
Gray fox	**99.64**	97.15	96.09	98.22	97.86	97.51	97.15	98.58
Moose	**96.76**	**96.76**	95.83	93.52	95.83	58.80	62.96	95.37
Mule deer	**98.49**	97.48	96.98	**98.49**	98.24	94.71	94.96	**98.49**
Armadillo ^5^	**100**	98.23	**100**	**100**	97.35	97.35	**100**	**100**
Raccoon	**99.20**	96.80	97.20	98.00	98.00	96.00	93.20	97.20
Red deer	92.25	91.98	91.44	95.19	**95.72**	87.97	86.36	92.78
Red fox	62.30	62.30	60.66	**75.41**	62.30	40.98	36.07	47.54
Hare ^6^	**99.62**	98.86	97.34	96.96	98.48	98.48	97.34	98.10
Skunk ^7^	99.18	99.18	98.77	99.59	**100**	98.77	98.35	99.59
Opossum ^8^	94.74	89.47	89.47	94.74	**100**	94.74	94.74	94.74
Wild boar	95.32	96.78	95.61	96.49	**97.08**	86.84	87.13	94.44
Wild turkey	98.06	96.13	98.71	**99.35**	**99.35**	87.74	82.58	96.77
Average	92.91	90.92	91.83	**94.78**	93.76	84.47	83.44	90.53

^1^ American animals; ^2^ Black-tailed jackrabbit; ^3^ California quail; ^4^ Eastern gray squirrel; ^5^ Nine-banded armadillo; ^6^ Snowshoe hare; ^7^ Striped skunk; ^8^ Virginia opossum.

**Table 3:** MCTI accuracy comparison.

Species or Parameter Number	DANAS (Ours)	MobileNet-v2 [48]	EfficientNet [49]	DenseNet [50]	Resnet-18 [51]	ResNext [52]	Wide_ResNet [53]	Random Search
Para. num.	1.43	2.25	4.04	6.98	11.19	23.02	66.88	**0.70**
Agouti	91.86	91.86	**94.19**	91.86	93.60	93.02	86.05	83.72
Bird	**97.02**	88.10	87.50	91.07	92.26	89.29	92.26	88.69
Agouti ^1^	**97.77**	86.16	86.61	92.41	87.05	90.18	91.96	92.86
Peccary ^2^	**90.12**	86.63	81.98	88.95	77.91	83.72	86.63	**90.12**
Opossum	94.42	96.14	93.56	**97.00**	**97.00**	94.42	96.14	93.13
Hare ^3^	**95.31**	66.41	75.78	88.28	92.19	82.03	86.72	70.31
Tinamou ^4^	73.74	65.66	74.75	75.76	75.76	68.69	**81.82**	41.41
Mouflon	93.86	88.60	81.58	**94.74**	89.47	89.47	88.60	76.32
Ocelot	**96.41**	88.02	89.82	89.22	92.22	91.62	90.42	92.81
Paca	90.71	89.29	90.71	**92.14**	90.00	91.43	**92.14**	78.57
Deer ^5^	**99.07**	96.26	96.26	98.60	98.60	97.20	96.73	94.39
Red Deer	**97.46**	91.86	95.93	96.69	**97.46**	96.95	96.69	94.40
Red Fox	99.76	99.29	99.06	99.76	**100**	62.59	61.18	97.88
Red Squirrel	99.80	98.04	98.82	**100**	99.80	97.45	99.61	99.21
Roe Deer	94.85	97.00	95.71	97.85	97.00	97.00	**98.71**	94.42
Spiny Rat	98.26	96.81	97.39	**98.84**	98.55	95.36	96.23	95.07
Coati ^6^	79.12	71.43	**81.32**	72.53	75.82	80.22	79.12	68.13
Deer ^7^	**96.72**	89.34	88.52	91.80	90.16	92.62	87.70	92.62
Wild Boar	**100**	**100**	**100**	**100**	**100**	**100**	**100**	93.47
Mouse ^8^	**100**	**100**	99.19	97.57	100	97.57	98.38	99.19
Average	**94.31**	89.34	90.43	92.75	92.24	89.54	90.35	86.84

^1^ Coiban agouti; ^2^ Collared peccary; ^3^ European hare; ^4^ Great Tinamou; ^5^ Red brocket deer; ^6^ White-nosed coati; ^7^ White-tailed deer; ^8^ Wood mouse.

**Table 4:** Test results on Jetson X2.

Species in NACTI-a	DANAS	Species in MCTI	DANAS
American black bear	97.94	Agouti	92.44
American marten	25.00	Bird	97.62
American red squirrel	98.67	Coiban Agouti	97.77
Black-tailed jackrabbit	98.60	Collared Peccary	90.12
Bobcat	97.36	Opossum	93.56
California quail	96.67	European Hare	95.31
Cougar	98.29	Great Tinamou	69.70
Coyote	95.55	Mouflon	93.86
Eastern Gray squirrel	100	Ocelot	94.01
Elk	99.24	Paca	91.43
Gray fox	99.29	Red Brocket Deer	99.07
Moose	97.22	Red Deer	97.96
Mule deer	97.98	Red Fox	99.76
Nine-banded armadillo	100	Red Squirrel	100
Raccoon	98.80	Roe Deer	94.85
Red deer	91.44	Spiny Rat	98.26
Red fox	63.93	White-nosed Coati	78.02
Snowshoe hare	99.24	White-tailed Deer	96.72
Striped skunk	99.59	Wild Boar	100
Virginia opossum	94.74	Wood Mouse	100
Wild boar	95.32	Average	94.02
Wild turkey	98.06		
Average	92.86		

**Table 5:** Comparisons with other search methods.

Method	Search (Seconds)	CIFAR-10		CIFAR-100		ImageNet-16-120	
		Validation	Test	Validation	Test	Validation	Test
REA [56]	0.03	91.56 ± 0.13	**94.35** ± 0.18	**73.15** ± 0.49	73.05 ± 0.56	46.08 ± 0.77	**46.08** ± 0.78
RS [57]	1.00	91.48 ± 0.12	94.08 ± 0.26	72.63 ± 1.09	72.44 ± 0.70	45.90 ± 0.58	45.64 ± 0.85
REINFORCE [45]	1.00	**91.70** ± 0.06	**94.35** ± 0.19	73.52 ± 0.30	73.43 ± 0.52	**46.49** ± 0.41	45.98 ± 0.72
BOHB [58]	6.12	88.52 ± 1.39	91.77 ± 1.30	62.62 ± 9.73	62.74 ± 9.79	33.43 ± 9.18	33.22 ± 9.51
DANAS (ours)	4.24	91.58 ± 0.17	94.28 ± 0.21	72.85 ± 0.64	72.71 ± 0.87	45.99 ± 0.56	45.75 ± 0.83

**Bold text**: optimal mean accuracies; underlined text: suboptimal mean accuracies.

## Data Availability

Dataset NACTI is available at http://lila.science/datasets/nacti. Dataset MCTI is available at https://lila.science/datasets/missouricameratraps.

## Appendix A

Since LSTM plays the role of a sampler, options to sample have to be converted to length-fixed vectors to feed the LSTM. This conversion is accomplished via a technique called word embedding, i.e., options are uniquely represented by plain words such as “conv 3 × 3” and each word is mapped to a row of a $\left|V\right|\times d$ matrix, where V denotes the set of unique words, $\left|V\right|$ is the number of unique words, and d is a hyper parameter. The word-embedding matrix is implemented as a standalone layer in our sampler, which means the matrix elements were learned during the search. To sample a network from our search space, LSTM starts with a random word from V, i.e., a random row of word-embedding matrix is fed to the input layer of LSTM. The data flow through LSTM and yield two tensors: “hidden states” and “cell states”. Hidden states are associated with the immediately previous sampling, and cell states are associated with long-term sampling found useful during training. Hidden states are then fed to some linear layer whose output corresponds to the sampling options. For example, suppose there are five different inputs and four different types; then there are two separate linear layers (matrices) of size h×5 and h×4, where h denotes the dimension of hidden states. If an operation input is desired, then the hidden states are only fed to the h×5 linear layer, which yields a five-element vector, and the resulting vector is fed to SoftMax function to produce five probabilities. The probabilities are then used to build a categorical distribution, and the input is sampled based on the built distribution.

Mathematically, suppose the last sampling results in hidden states $h_{t-1}$; then, for tth sampling, LSTM attempts to sample blocks of the normal cell. For the jth block, LSTM firstly samples the number of operations in the block and then samples inputs and types of operations, namely, $h_{t-1}$ is fed to LSTM to produce $h_{t}$, and $h_{t}$ is fed to a linear layer. The linear layer produces a vector that is converted to probabilities via softmax(), and an integer $n_{j}$ is sampled based on the probability $P\left(n_{j}\left|\theta \right.\right)$ via categorical distribution. Thus, current block has $n_{j}$ operations. Starting from the first operation, $h_{t}$ is fed to LSTM to produce $h_{t+1}$ which is fed to a linear layer, and the vector produced by the linear layer is converted to probabilities. The operation input $a_{1}$ is then sampled based on $P\left(a_{1}\left|n_{j},\theta \right.\right)$, and $h_{t+1}$ is fed to LSTM to yield $h_{t+2}$, which is used to produce the probabilities to sample the operation type $a_{2}$ based on $P\left(a_{2}\left|a_{1},n_{j},\theta \right.\right)$. For the kth sampling, $h_{t+k-1}$ is fed to LSTM to yield $h_{t+k}$ which is fed to a linear layer to sample the option $a_{k}$ based on $P\left(a_{k}\left|a_{1\;:\left(k-1\right)},n_{j},\theta \right.\right)$. This sampling procedure continues until all blocks have been sampled. Then, LSTM starts to sample blocks of the reduction cell.

Specifically, suppose there are $n_{C}$ cells to sample; for the ith cell, LSTM needs to sample $B_{i}$ blocks. For the jth block, suppose LSTM samples the operation number $n_{j}$ based on $P\left(n_{j}\left|\theta \right.\right)$; then, LSTM needs to sample inputs and types for every $n_{j}$ operations. For the kth operation, suppose LSTM samples the input and type denoted by $a_{k}$ based on $P\left(a_{k}\left|a_{1\;:\left(k-1\right)},\theta \right.\right)$; then, the samplings related with the kth operation may be quantitatively represented by

(A1) $$\overset{n_{j}}{\underset{k}{\sum }}\log\left(P\left(a_{k}\left|a_{1\;:\left(k-1\right)},\theta \right.\right)\right),$$

and the samplings related with the jth block may be quantitatively represented by

(A2) $$\log\left(P\left(n_{j}\left|\theta \right.\right)\right)+\overset{n_{j}}{\underset{k}{\sum }}\log\left(P\left(a_{k}\left|a_{1\;:\left(k-1\right)},\theta \right.\right)\right),$$

and the samplings related with the ith cell may be quantitatively represented by

(A3) $$\overset{B_{i}}{\underset{j}{\sum }}\left(\log\left(P\left(n_{j}\left|\theta \right.\right)\right)+\overset{n_{j}}{\underset{k}{\sum }}\log\left(P\left(a_{k}\left|a_{1\;:\left(k-1\right)},\theta \right.\right)\right)\right).$$

Finally, the samplings related with $n_{C}$ cells may be quantitatively represented by

(A4) $$a_{\Sigma }\left(\theta \right)=\overset{n_{C}}{\underset{i}{\sum }}\overset{B_{i}}{\underset{j}{\sum }}\left(\log\left(P\left(n_{j}\left|\theta \right.\right)\right)+\overset{n_{j}}{\underset{k}{\sum }}\log\left(P\left(a_{k}\left|a_{1\;:\left(k-1\right)},\theta \right.\right)\right)\right).$$

Thus, the value of $a_{\Sigma }\left(\theta \right)$ is associated with θ of LSTM, and θ can be optimized via some optimization algorithm such as SGD. Even so, $a_{\Sigma }\left(\theta \right)$ alone cannot make SGD work because $a_{\Sigma }\left(\theta \right)$ contains no information about rewards, i.e., the gradient $\nabla_{\theta }a_{\Sigma }$ used in SGD is out of control since we are not sure whether $\nabla_{\theta }a_{\Sigma }$ leads to a promising position of high reward or not. This can be alleviated by introducing rewards, as described in the paper.
